# Cross-Linked Enzyme Aggregates and Their Application in Enzymatic Pretreatment of Microalgae: Comparison Between CLEAs and Combi-CLEAs

**DOI:** 10.3389/fbioe.2021.794672

**Published:** 2021-12-09

**Authors:** Cristina Blanco-Llamero, Paz García-García, Francisco Javier Señoráns

**Affiliations:** Healthy Lipids Group, Departmental Section of Food Sciences, Faculty of Sciences, Universidad Autónoma de Madrid, Madrid, Spain

**Keywords:** CLEAs, combi-CLEAs, immobilization, microalgae, enzymatic pretreatment, Viscozyme®, Alcalase®, Celluclast®

## Abstract

Carrier-free immobilization is a key process to develop efficient biocatalysts able to catalyze the cell wall degradation in microalgae where the traditional solid supports cannot penetrate. Thus, the insolubilization of commercial Celluclast®, Alcalase®, and Viscozyme® enzymes by carrier-free immobilization and their application in microalgae pretreatment was investigated. In this study, different precipitants at different ratios (ethanol, acetone, and polyethylene glycol 4000) were tested in the first part of the method, to establish the precipitation conditions. The screening of the best precipitant is needed as it depends on the nature of the enzyme. The best results were studied in terms of immobilization yield, thermal stability, and residual activity and were analyzed using scanning electron microscopy. Moreover, a novel strategy was intended including the three enzymes (combi-CLEAs) to catalyze the enzymatic degradation of *Nannochloropsis gaditana* microalgal cell wall in one pot. The carrier-free immobilized derivatives were 10 times more stable compared to soluble enzymes under the same. At the best conditions showed its usefulness in the pretreatment of microalgae combined with ultrasounds, facilitating the cell disruption and lipid recovery. The results obtained suggested the powerful application of these robust biocatalysts with great catalytic properties on novel and sustainable biomass such as microalgae to achieve cost-effective and green process to extract valuable bioactive compounds.

## 1 Introduction

Nowadays, microalgae have gained attention as an alternative biomass due to a wide range of characteristics that make them an interesting biomass source. Among these, it is interesting to highlight their ability to rapidly accumulate important amounts of added value components. In addition, their cultivation does not require arable lands or pure water; indeed, they are able to grow in wastewater and in bioreactors, which make microalgae a promising and environment-friendly source of bioactive compounds such as lipids, carotenoids, and proteins. Moreover, the composition of microalgae highly varies among the different species, which make them more versatile. On its own, *Nannochloropsis gaditana* is described as a microalga able to produce important amounts of lipids, including polar lipids, omega-3 polyunsaturated fatty acids, and carotenoids such as carotenes and xanthophylls, which have benefits for human health widely recognized (Yen et al., 2013; Gong and Bassi, 2016; Castejón et al., 2018; Castejón and Señoráns, 2019; Punia et al., 2019; Zhang et al., 2019; Castejón and Señoráns, 2020; Katiyar and Arora, 2020; Señoráns et al., 2020; Blanco-Llamero and Señoráns, 2021).

Regarding extraction step, the most critical point is usually the selection of an appropriate extraction technique due to the presence of a dense and firm cell wall in microalgae, which difficult the extraction of bioactive compounds from the cells. *N. gaditana* cell wall contains mainly proteins and other biopolymers such as cellulose or pectin, among others. Thus, the cell wall must be properly disrupted to efficiently recover intracellular bioactive compounds (Blanco-Llamero et al., 2021). Several methods for cell disruption could be applied. Enzymatic digestion before extraction facilitates both lipid extraction and downstream process of microalga biomass. Weakened cell walls reduce solvent and energy inputs needed for extraction. Because the microalgae cell wall includes different fibers and proteins, an enzymatic cocktail with different hydrolytic activities like carbohydrases and proteases should be applied for this purpose. Thus, the use of enzymes has demonstrated to facilitate the hydrolysis of microalgae cell walls, which could be increased if the enzymes are combined between each other and with other physical disruption methods, obtaining increased oil yields. As it has been proved in previous studies on *N. gaditana* dry biomass, the combination of different enzymes, such as Viscozyme® (containing a wide range of carbohydrases, including arabinase, cellulase, beta-glucanase, hemicellulase, and xylanase), Celluclast® (containing cellulase), and Alcalase® (a protease cocktail) with other physical methods, enhance and optimize polar lipid recovery. Indeed, *N. gaditana* cell wall has been reported to be extremely rigid, composed by a structure which difficult the action of the enzymes; thus, it has been demonstrated the need to combine enzymes, both proteases and carbohidrases to successfully break microalgae cell wall, and even this way, the process continues being challenging. The physical methods such as ultrasounds (USs) accelerate the action of the enzymes, showing a synergic effect of both pretreatments, enzymatic and US, on the break of *N. gaditana* cell wall. When each pretreatment was applied separately on the microalgae biomass the oil yield was fewer than the one obtained by their combination, obtaining an effective combined pretreatment previously optimized in other works of the research group (Zuorro et al., 2016; Chen et al., 2017; Zhang et al., 2017; Zhang et al., 2018a; Dixon and Wilken, 2018; Wang et al., 2019; Blanco-Llamero et al., 2021).

The major problem while using soluble enzymes is the production cost, because they are not reusable and are highly unstable under different conditions of microalgae pretreatment, which make it necessary to use high amounts of commercial enzymes. Immobilization is a well-documented alterative for enzyme recycling, improve stability, and reduce the loss of activity under operational conditions of the process. Several approaches for enzyme immobilization can be find in the literature such as adsorption, covalent attachment, entrapment, encapsulation, and cross-linking (Gao et al., 2021); (Chen et al., 2018).

**FIGURE 1:**
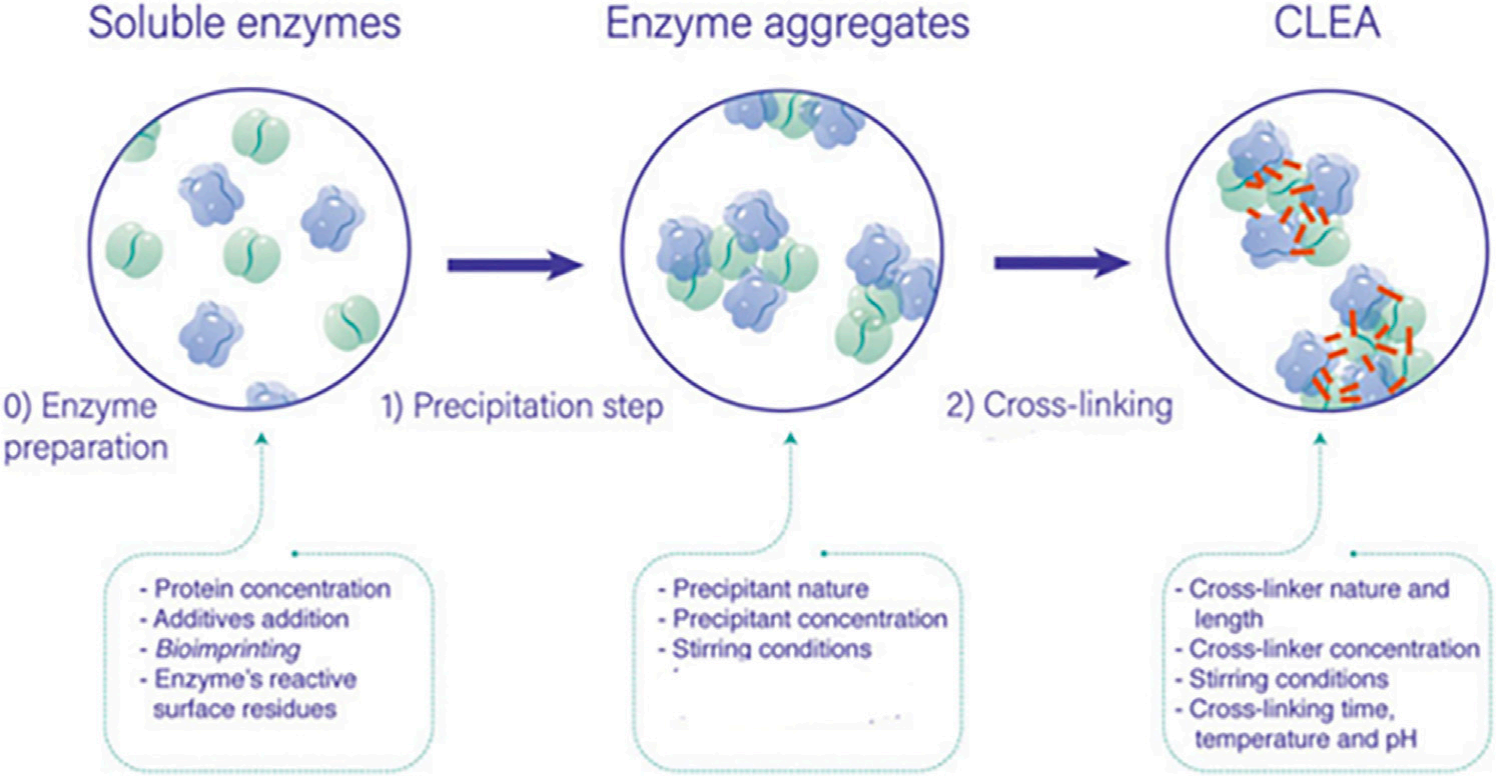
General process for the preparation of cross-linked enzyme aggregates (CLEAs) and the possible variables governing its final performance (Velasco-Lozano, 2020).

Carrier-free cross-linked enzyme aggregates (CLEAs) are one of the immobilization methods, which has attracted attention due to ease of preparation and robustness of immobilized enzymes without the need of support (Figure 1). CLEAs are prepared in several steps; a first one of enzyme aggregation is produced by mixing them with the precipitant agent (e.g., ammonium sulfate, organic solvents, or polymers) in aqueous solutions, in which enzymatic solutions have to contain a determined protein concentration to produce the aggregates. If the enzymatic solution does not contain enough protein concentration, then protein feeders such as bovine serum albumin (BSA) or other protein can be added to facilitate enzyme precipitation and CLEA handling. The second step during CLEA preparation involves the irreversible insolubilization of the enzyme aggregation by a cross-linking procedure. There are various cross-linking agents described in the literature, among which glutaraldehyde (GA) is the most employed one because of its high reactivity, availability, and low price. When GA is the cross-linker, enzyme aggregates are irreversibly bonded through covalent bonds between its free amino groups (mainly from lysine residues) and both aldehyde moieties of GA, yielding an insoluble biocatalyst with high stability and activity. It must be emphasized the importance of the cross-linking agent because it prevents the leaching of the enzyme and can cause steric hindrance. In this work, the effect of the cross-linker was not studied, and GA was used for CLEA production, as it was previously optimized in other studies. On the contrary, the action of the precipitant agent was deeply studied due to the potential interaction of the different enzymes with them due to their different structures (Velasco-Lozano, 2020; Hero et al., 2020; Xu et al., 2020; Bilal et al., 2021; Guajardo et al., 2021).

On the other hand, the use of combi-CLEAs (co-immobilization of two or more enzymes in a single CLEA for the sole purpose of performing two or more sequential biotransformations) reduces the cost of production at industries and produces better yield as compared to multi-step reactions integrating them into a one-pot process. They also have the benefit of fewer unit operations, less solvent, shorter cycle times, and less waste, resulting in economic and environmental advantages. Furthermore, one-pot reactions can be used to achieve equilibria toward product and biomass, which may be a high advantage in enzymatic procedures. (Perwez et al., 2021).

In the present work, CLEA and combi-CLEA containing Viscozyme®, Alcalase®, and Celluclast® have been developed and compared with soluble free enzymes regarding thermal and pH stability, to then be applied in microalgae aqueous pretreatment combined with USs to enhance lipid recovery from biomass.

## 2 Material and Methods

### 2.1 Materials

*Nannochloropsis gaditana* dry biomass was provided by Cianoalgae SL. (Madrid, Spain). Absolute ethanol (PRS grade), acetone, polyethylene glycol (PEG) 4000, sodium hydrogen carbonate, and potassium hydroxide were purchased from Panreac Química S.A (Barcelona, Spain). The water used was Milli-Q grade (Millipore Sigma, Burlington, MA, United States). Viscozyme® from *Aspergillus aculeatus* containing a wide range of carbohydrases, including arabinase, cellulase, beta-glucanase, hemicellulase, and xylanase; Celluclast® from *Trichoderma reesei* containing cellulase; and Alcalase® were kindly donated by Novozymes (Bagsvaerd, Denmark). The substrate Boc-Ala-Onp (NPA), 3,5-Dinitro-2-hydroxybenzoic acid (DNS), carboxymethylcellulose sodium salt (CMC), potassium sodium tartrate tetrahydrate (Rochelle salts), acetonitrile, and GA solution 25% were purchased from Sigma (Burlington, MA, United States). All other reagents and solvents used were of analytical or HPLC grade.

### 2.2 Bradford Method for Protein Quantification

The protein concentration was determined by Bradford (Bradford, 1976). The samples were diluted (1/2, 1/5, 1/10, and 1/20) to obtain different enzymatic solutions. To perform the measurements, 20 μl of the sample was added to 1 ml of Bradford’s solution and allowed to react for 30 min. The absorbance was measured at 595 nm on a model UV-Vis UV-1280 spectrophotometer. The absorbance range of the samples must be between 0.1 and 1 for measurements to be reliable. Different concentrations were obtained from a known standard curve for BSA. Protein determinations were performed at least in duplicate in all cases.

### 2.3 Spectrophotometric Assays

#### 2.3.1 Determination of Alcalase® Activity by NPA Hydrolysis

The determination of enzyme activity was carried out by using the substrate Boc-Ala-Onp (NPA), according to the methodology presented in other works (Kimberle et al., 2020). The hydrolysis of the substrate (100 mM NPA, prepared in acetonitrile) was conducted at pH 7 (50 mM sodium phosphate buffer, containing 20% of ethanol) and 25°C. The product formation was quantified in a spectrophotometer (405 nm), and the enzyme activity was expressed in UNPA (1UNPA = amount of enzyme that was capable of hydrolyzing 1 μmol of substrate per minute at pH 7 and 25°C). The immobilization yield in terms of activity was defined as the expressed activity of the biocatalyst with respect to the initial activity offered. Initial activity of commercial Alcalase® was 2.4 U/g.

#### 2.3.2 Determination of Celluclast® and Viscozyme® Activity

Several methods exist for assaying cellulase enzyme activity. Among those, carboxymethyl-Cellulase “CMCase” was used in this research. Celluclast® and Viscozyme® activity was measured by dinitrosalicylic acid method (DNS), using glucose as a standard and measuring its absorbance by UV-Vis spectroscopy at 540 nm (Romero-Fernández et al., 2018). For DNS preparation, 200 ml of water under stirring was heated until 100°C; once the temperature was achieved, 10 g of DNS were added slowly. Once the mixture became homogeneous, 16 g of NaOH were added, and finally, 300 g of Rochelle salts. The mixture was made up to 1 L with water.

The activity assay was performed as follows: 0.8 ml of substrate (CMC) was mixed with 0.2 ml of commercial enzyme, and they were incubated for 10 min. After this time, 1 ml of the prepared DNS was added, boiled for 5 min, and cool down in iced water. Water (9 ml) was added to each sample, and the absorbance was measured at 540 nm to study the glucose concentration that was liberated. One unit of enzyme activity was defined as the amount of enzyme required to release 1 µmol of reducing sugars in 1 min (Zuorro et al., 2016; Chen et al., 2018; Poorakbar et al., 2018). The activity of commercial Viscozyme® and Celluclast® was 100 and 700 U/g, respectively.

### 2.4 CLEAs and Combi-CLEAs Formation

#### 2.4.1 Protein Concentration

For CLEA production, it is recommendable to work with protein concentrations between 2 and 100 mg/ml. In this work, after protein quantification of commercial enzymes by Bradford, CLEAs and combi-CLEAs were performed in all cases using a final concentration of 20 mg/ml. For single CLEAs, 20 mg/ml of each enzyme was prepared in separated carrier-free derivatives. In the case of combi-CLEAs, a mixture containing Viscozyme®, Celluclast®, and Alcalase® (one third of each commercial enzyme to a final concentration of 20 mg/ml) was used.

#### 2.4.2 Screening of Different Precipitants in the First Step of Single CLEA Formation

In the present work, three different precipitants were studied to test the distinct interaction between precipitants and enzymes to develop the first step in CLEA formation. Two organic solvents (ethanol and acetone) and one polymer (PEG 4000) were studied at different ratios (60%–90% and 50%–90%, respectively). The three precipitants were all studied in combination with the three commercial enzymes (Viscozyme®, Celluclast®, and Alcalase®), developing single CLEAs of each enzyme separately.

The first step in CLEA formation (precipitation) was developed as follows: 20 mg/ml of enzymatic solution (the corresponding volume was determined for each commercial enzyme taking into account the protein concentration) was mixed with precipitant agent (prepared at different ratios in distilled water) in a final volume of 4 ml. The mixture was gently mixed at room temperature during 15 min until the solution appeared visibly turbid.

#### 2.4.3. Cross-Linking With Glutaraldehyde in the Second Step of Single CLEA Formation

In this step, GA was immediately added to the solutions to obtain a defined concentration of 75 mM (20 µl of GA solution 25%) and vigorously mixed by vortexing for 1 min. The mixtures were then incubated during20 h at 4°C under vigorous agitation. After cross-linking time, the preparations were centrifuged at 5,000 g for 30 min at 4°C, and the supernatant was discarded (containing enzymes that did not react). To resuspend and wash the CLEAs, 5 ml of buffer solution was added and mixed by vortexing for 5 min and centrifuged again. The wash step was repeated three times to remove traces of reagent. Finally, the CLEAs were resuspended in the buffer solution at a proper concentration to assay their enzymatic activity spectrophotometrically and their protein concentration by Bradford to calculate the immobilization yield and compare them (Velasco-Lozano, 2020; Zhang et al., 2018b). The immobilization yield of CLEAs was calculated by the ratio of enzyme units (calculated by the protein in the supernatant) to the total unit of enzymes loaded for immobilization.

#### 2.4.4 Combi-CLEA Formation Under Optimal Conditions

Once the optimized conditions for CLEA formation for different enzymes assayed were obtained using different precipitants (acetone, ethanol, and PEG), they were used to obtain combi-CLEAs. The protocol was the same as for the separated CLEAs. Briefly, the enzymatic mixture containing the three enzymes at final concentration of 20 mg/ml was mixed with precipitants in separated experiments (acetone at 90%, ethanol at 90%, and PEG at 80%) and incubated at room temperature under stirring for 15 min. Then, GA was added and incubated overnight at 4°C under vigorous stirring. After that, mixtures were centrifuged, and combi-CLEAs were obtained in the pellet (supernatant was discarded). Combi-CLEAs were washed three times to remove traces of reagents. Immobilization yield was obtained in the same was as for the separated CLEAs.

### 2.5 Inactivation Assays of Carrier-Free Biocatalysts

The determination of biocatalysts stability was carried out at different temperatures (50°C and 55°C) and different pH (25 mM phosphate buffer at pH 7 and acetate buffer at pH 5.0), containing the same amount of protein to compare the results. Aliquots were withdrawn for 24 h. Each sample was analyzed at different times by measuring its enzymatic activity as described above. Residual activity was calculated as the ratio between activity at a given time and the activity at the beginning of incubation. Half-life times (time in which the residual enzyme activity is half of its initial value) were calculated as previously described in the literature (Romero et al., 2012). Stabilization factors were determined by comparing half-life times of different derivatives and a reference.

### 2.6 Enzymatic Pretreatment of Microalgae Using CLEAs and Combi-CLEAs

The optimum CLEAs and combi-CLEAs obtained were applied to microalgae using the pretreatment described below and compared to soluble enzymes in terms of oil yield extracted [using the Folch method (Folch et al., 1957)] and microalgal cell wall disruption rate studied by scanning electron microscopy (SEM).

The protocol of the optimized enzymatic pretreatment of *N. gaditana* biomass was previously described in literature (Blanco-Llamero et al., 2021). One gram of dry microalgal biomass was resuspended in 10 ml of sodium citrate buffer 0.1 M pH 5.0 containing soluble enzymes (20 mg/ml containing the three soluble commercial enzymes), single CLEAs (containing 20 mg/ml of each enzyme in the separated CLEAs), or combi-CLEAs (combined CLEAs containing a mixture of the three commercial enzymes at a final concentration of 20 mg/ml) per gram of biomass. In addition, 1 g of biomass was resuspended in a 10 ml buffer of without enzymes (blank solution). US-assisted enzymatic pretreatment was carried out with a US bath Elmasonic S40H Elma brand (Singen, Germany) with automatic control of time (6 h) and temperature (55°C), US frequency of 37 kHz, and bath power of 140 W.

The flask content was centrifuged at 3,000 rpm for 10 min, the supernatant was discarded, and pellet biomass was kept at 4°C for its extraction by the Folch method and characterization. Experiments were performed at least in triplicate in all cases.

### 2.7 SEM-EDX Analyses of CLEAs

To envisage surface morphologies, images of CLEAs were captured using SEM (Hitachi, TM-1000). For observing CLEA particles under SEM energy-dispersive X-ray (SEM-EDX) analytical system, a droplet of CLEA sample was dispensed on a polycarbonate track-etched membrane, freeze-dried, and platinum-coated using a sputtering coater. Structural characterization of CLEAs and pretreated microalgae biomass were analyzed.

### 2.8 Statistical Analysis

The results were expressed as the mean of the experiments and its SD.

Statistical analysis was performed in the SISA (Simple Interactive Statistical Analysis) online software available at http://www.quantitativeskills.com/sisa/statistics/ttest.htm (accessed August 20, 2021). The data were subjected to a t-test to examine whether the two groups mean differ from one another. To test whether there is an overall statistically significant difference between three or more means, the data were subjected to a one-way analysis of variance using the F-test for discrimination between means (*p* < 0.05).

## 3 Results and Discussion

### 3.1 Development of Alcalase®-CLEAs

#### 3.1.1 Screening of Different Precipitants for CLEA Production

The first step during CLEA preparation consists in the formation of active enzyme aggregates. To this aim, a preliminary screening of non-denaturant precipitants was conducted by testing the precipitant efficiency and the residual activity of the precipitated enzyme expressed in Table 1.

**TABLE 1:** Immobilization performance of Alcalase®-CLEAs at different precipitant conditions.

Precipitant type	Precipitant concentration (%)	Immobilization yield (%)	Immobilized protein (mg)	Enzymatic activity of immobilized Alcalase (U/g)
Ethanol	60	76.67 ± 0.26	15.33 ± 0.30	1.33 ± 0.21
	70	82.87 ± 0.31	16.57 ± 0.39	1.39 ± 0.11
	80	90.38 ± 0.40	18.08 ± 0.27	1.81 ± 0.32
	90	96.65 ± 0.16	19.33 ± 0.81	2.22 ± 0.24
Acetone	60	93.47 ± 0.18	18.69 ± 0.13	1.97 ± 0.30
	70	94.73 ± 0.22	18.95 ± 0.52	2.32 ± 0.17
	80	98.71 ± 0.61	19.74 ± 0.14	2.14 ± 0.22
	90	98.28 ± 0.66	19.66 ± 0.26	2.26 ± 0.10
PEG	50	92.45 ± 0.56	18.49 ± 0.32	2.26 ± 0.31
	60	87.82 ± 0.39	17.56 ± 0.61	2.33 ± 0.08
	70	86.40 ± 0.20	17.28 ± 0.25	1.93 ± 0.19
	80	87.82 ± 0.78	17.56 ± 0.42	1.94 ± 0.09
	90	83.19 ± 0.85	16.64 ± 0.36	1.75 ± 0.23

Thus, in a first set of experiments, the cross-linking immobilization of Alcalase® was explored. Several parameters need to be considered, such as the selection of the precipitating agent, the ratio of the precipitant, and the need or not of adding BSA to achieve a biocatalyst with good physical characteristics (to be recovered after centrifugation) with high activity and high immobilization performance. The effect of precipitant type and ratio on activity recovery and formation of CLEAs was evaluated. Acetone and ethanol (organic solvents) and PEG 4000 and PEG at different concentrations (60%–90% and 50%–90%, respectively) (non-ionic polymers) were studied at 20°C and using GA as cross-linking agent. The immobilization yield was tested in terms of protein concentration determined by Bradford and in terms of enzyme activity with spectrophotometric activity assay to corroborate the results. Results are detailed in Table 1 and expressed as the average between both immobilization yields.

The best percentage of immobilized Alcalase® was achieved in the case of acetone, which ranged from 93% to 98% for all the ratios tested, followed by ethanol, which varied from 76% to 96%. In general, the immobilization yield increased when the concentration of the organic precipitant grew. On the other hand, taking PEG as a precipitant agent, it appeared to follow the opposite trend than ethanol and acetone, and the immobilized percentage increased as the PEG concentration decreased, either taking into account protein concentration or enzyme activity. This result is in agreement with previous works where amounts around 50% of PEG were shown to produce huge and stable molecular aggregates, whereas higher percentages provoke distortions in the enzyme structure leading to non-stable aggregates (García-García et al., 2021). Interestingly, PEG at 50% of concentration resulted in slightly higher results than the 60% conditions. Thus, both conditions were selected to be further investigated in the stability test.

In summary, combining both analyses (immobilization yield and recovered activity), the best precipitant agents were ethanol at 90%, acetone at 90% and 70%, and PEG at 50% and 60%. Ethanol at 90% was selected due to its higher activity and immobilization yield, compared to the other precipitant conditions. Although acetone at 90% led to higher immobilization rate, its enzymatic activity resulted to be lower, so acetone at 70% was also selected. Moreover, PEG at 50% and 60% was also chosen. These conditions were selected to be tested in the inactivation assays, and this way selects the final conditions.

#### 3.1.2 Kinetic Characterization of Free Alcalase® and Alcalase®-CLEAs by Inactivation Assays

To further compare between different immobilization conditions (ethanol at 90%, acetone 90% and 70%, and PEG 50% and 60%), stability assays were performed in different temperature and pH conditions comparing free enzymes and CLEAs. The conditions were chosen in regard to the optimum conditions of previous works on *N. gaditana* pretreatment using the combination of the three enzymes (Mahdy et al., 2014; Safi et al., 2017; Blanco-Llamero et al., 2021), using this model as a proof of concept of the derivatives activity on microalgae and taking it as the final objective of the present study.

**FIGURE 2:**
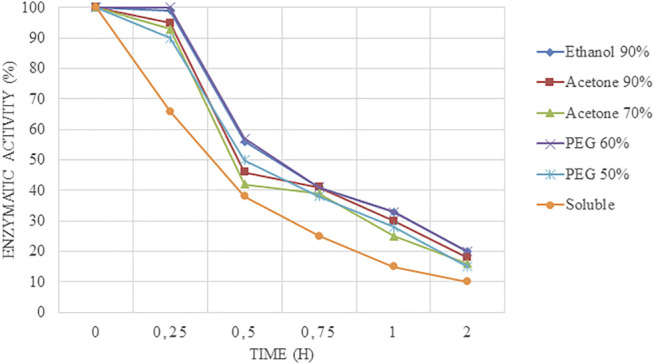
Time course of inactivation at 55°C and pH 5.0 at different times of Alcalase®-CLEAs using PEG, ethanol, and acetone as precipitants.

It is interesting to point out that CLEAs produced were more stable than the soluble enzymes because the derivatives facilitate the stabilization of the enzyme by creating a strong bonding between the amino groups of the enzyme molecule and GA. Furthermore, the structural organization of CLEAs drastically avoids conformational modifications, resulting in higher pH tolerance than free enzymes. Stability assays at 55°C and pH 5.0 (Figure 2) revealed that free soluble enzyme half-life was achieved before 30 min of assay. On the other hand, CLEAs tested maintained the total activity for 15 min, and then, it started to decrease dramatically. This fact could be attributed to the aggressive conditions tested because at medium-high temperature, each degree could be critical for the integrity of the enzyme; thus, lower temperature (50°C) was tested at pH 5.0.

**FIGURE 3:**
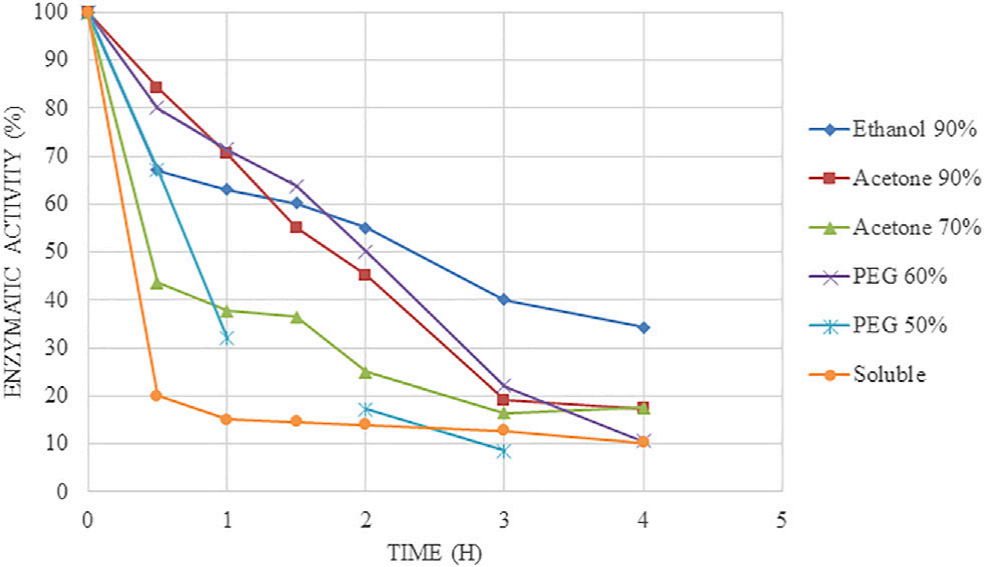
Time course of inactivation at 50°C and pH 5.0 at different times of Alcalase®-CLEAs using PEG, ethanol, and acetone as precipitants.

Considering 50°C and pH 5.0 (Figure 3), all the CLEAs and the soluble Alcalase® increased their stability compared to 55°C, as expected. There were clear differences between acetone at 70% and 90%, which were similar in terms of immobilization percentage. Acetone at 70% lost 50% of its initial activity at 30 min, whereas acetone at 90% achieve this level at 2 h. Nevertheless, both of them only retains 20% of its initial activity at 4 h. In addition, it could be seen big differences between PEG at different rates, showing better stability 60% compared to 50% (Sheldon, 2006; Bilal et al., 2021).

On the other hand, ethanol at 90% resulted to be the biocatalyst with the best stability, maintaining 50% of activity at 4 h, whereas the other CLEAs only retained 20% of its initial activity at this stage. As it can be seen in Figure 3, the kinetic of inactivation of the CLEA was less dramatic with ethanol at 90% than compared to the other conditions. With regard to the soluble enzyme, its kinetic of inactivation was very rapid, with a half-life time of less than 30 min. This result compared to that obtained for CLEAs in ethanol at 90% results in a derivative 10 times more stable than the soluble enzyme.

**FIGURE 4:**
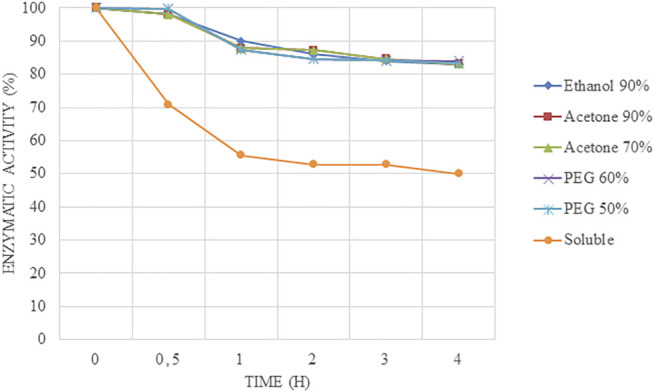
Stability results at 50°C and pH 7.0 at short times of Alcalase®-CLEAs using PEG, ethanol, and acetone as precipitants.

When carrier-free derivatives were tested at pH 7.0 and 50°C to test a neutral pH as well (Figure 4), no differences were found between the different immobilized biocatalysts (acetone-Alcalase®-CLEAs, PEG-Alcalase® CLEAs, and ethanol-Alcalase®-CLEAs) because they retained over 80% of their relative activities without differences between them. However, the free enzyme lost 50% of its relative activity at 2 h.

Thus, immobilization of Alcalase® in CLEAs results in a remarkably improvement in temperature resistance due to biocatalyst rigidity attributed to the stabilization of three-dimensional structure of the protein, which contributes to higher activity than the free enzymes due to conformational changes in enzymes structure.

### 3.2 Development of Celluclast®-CLEAs

The results obtained from the Celluclast®-CLEA production (Table 2) in terms of protein concentration and enzymatic activity of the derivative mostly coincided with the ones resulted from the Alcalase®-CLEAs, because the best conditions for acetone and ethanol were both 90% ratio with a remarkably high protein loaded in both cases (99%). However, the results also had some differences from the previous ones, because CLEAs were not formed at all of the conditions tested. In fact, ethanol at lower ratios was not able to produce visible CLEAs, so occurs with acetone at the lowest ratio tested (60%). These differences could be attributed to the different enzymatic structure of Alcalase® and Celluclast®, producing different interaction with the precipitant agents. This fact highlights the importance to optimize immobilization conditions for each enzyme separately. On the other hand, all of the ratios tested for PEG as a precipitant agent obtained similar immobilization results.

**TABLE 2:** Immobilization performance of Celluclast®-CLEAs at different precipitant conditions.

Precipitant type	Precipitant concentration (%)	Immobilization yield (%)	Immobilized protein (mg)	Enzymatic activity of immobilized Celluclast (U/g)
Ethanol	90	99.20 ± 0.12	19.84 ± 0.10	679.26 ± 0.85
Acetone	70	98.25 ± 0.14	19.65 ± 0.11	561.73 ± 0.47
	80	98.70 ± 0.06	19.74 ± 0.10	648.15 ± 0.55
	90	99.38 ± 0.48	19.87 ± 0.31	677.53 ± 0.48
PEG	50	95.05 ± 0.37	19.01 ± 0.20	566.91 ± 1.21
	60	96.36 ± 0.18	19.27 ± 0.15	509.88 ± 1.03
	70	97.67 ± 0.62	19.53 ± 0.31	573.83 ± 0.95
	80	98.64 ± 0.34	19.72 ± 0.26	658.52 ± 0.86
	90	95.87 ± 0.52	19.17 ± 0.38	623.26 ± 0.77

It is interesting to highlight the higher immobilization recovery obtained in this work compared with previous works on CLEAs with other enzymes, in which the best immobilization rate did not go up from 30% to 70% for PEG, ammonium sulfate, n-propanol, acetone, and tert-butanol as precipitant agents, whereas in the present study, the results up to 80% were obtained for all the enzymes studied (Bilal et al., 2021; Xu et al., 2020; Perwez et al., 2021).

Immobilization rates were also studied in terms of enzymatic activity for Celluclast® and Viscozyme® (data not shown), which coincided with the results obtained in terms of protein concentration shown in Table 2.

### 3.3 Development of Viscozyme®-CLEAs

Viscozyme®-CLEAs results were the ones that differed the most from the other ones (Table 3), which may be due to the fact that Viscozyme® is already an enzymatic cocktail containing different enzymes and generating different interactions with the materials tested. Indeed, these CLEAs could be considered already combi-CLEAs because different enzymes were immobilized successfully obtaining visible CLEAs. Analyzing results deeper, the immobilized percentage for all the conditions was slightly lower than the one obtained with the previous enzymes, which could be explained because it is more difficult to immobilize more enzymes compared to one due to the complex interaction that could appear between all of them. Although, the percentages achieved could be considered as high ones compared to previous works on CLEAs with other enzymes (Bilal et al., 2021; Xu et al., 2020; Perwez et al., 2021).

**TABLE 3:** Immobilization performance of Viscozyme®-CLEAs at different precipitant conditions.

Precipitant type	Precipitant concentration (%)	Immobilization yield (%)	Immobilized protein (mg)	Enzymatic activity of immobilized Viscozyme (U/g)
Acetone	60	80.01 ± 0.18	16.02 ± 0.20	75.56 ± 0.82
	70	77.41 ± 0.20	15.48 ± 0.22	72.84 ± 0.47
	80	90.48 ± 0.56	18.09 ± 0.48	89.04 ± 1.01
	90	86.05 ± 0.58	17.21 ± 0.57	81.98 ± 0.88
Ethanol	60	86.90 ± 0.17	17.38 ± 0.13	80.25 ± 0.93
	70	79.90 ± 0.12	15.98 ± 0.10	80.99 ± 0.28
	80	82.07 ± 0.16	16.41 ± 0.22	72.84 ± 0.77
	90	87.02 ± 0.43	17.40 ± 0.41	89.04 ± 0.72
PEG	50	83.28 ± 0.55	16.85 ± 0.60	80.75 ± 0.14
	60	85.17 ± 0.42	17.03 ± 0.50	80.25 ± 0.90
	70	91.02 ± 0.39	18.20 ± 0.51	90.05 ± 0.52
	80	91.51 ± 0.23	18.30 ± 0.26	90.33 ± 0.35
	90	94.69 ± 0.31	18.93 ± 0.28	91.36 ± 0.72

The best immobilization conditions for Viscozyme® resulted to be acetone at 80% and 90% and ethanol at 90% as occurred with the other enzymes and PEG at all the ratios tested.

### 3.4 Development of Combi-CLEAs Under Optimized Process Parameters

**FIGURE 5:**
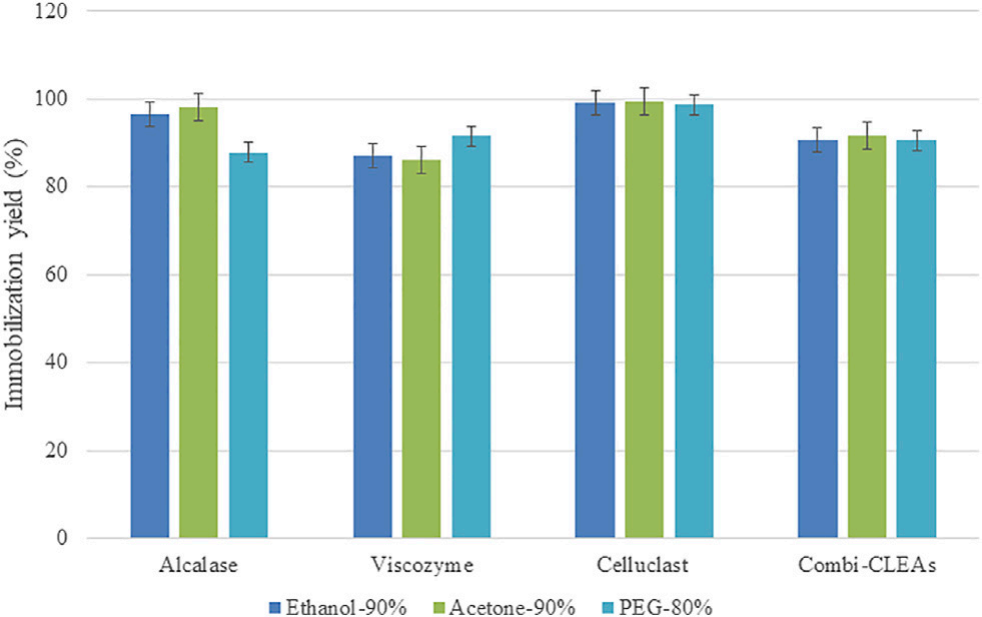
Immobilization performance of CLEAs and combi-CLEAs at best precipitant conditions.

Combi-CLEAs were produced mixing the three enzymes at a proper concentration (final concentration of 20 mg/ml) before adding the precipitant agent and following the same method as the one used for CLEAs of each enzyme. On the basis of the obtained results, combi-CLEAs including Alcalase®, Celluclast®, and Viscozyme® were intended, employing the best conditions for the three enzymes and using compromise conditions when it was needed. The chosen conditions resulted to be acetone at 90%, ethanol at 90%, and PEG at 80%. Combi-CLEAs were produced successfully with interesting immobilization rates (over 90%). Immobilization performance was similar for the three derivatives (Figure 5).

Stability of the best combi-CLEAs obtained was also investigated employing the stability conditions that resulted more illustrated previously (50°C and pH 5.0) (data not shown). It is interesting to highlight the fact that the combi-CLEAs was less stable than the CLEAs of each enzyme separately, as it was expected because of the complexity of the derivatives. Even so, the combi-CLEAs were remarkably more resistant than the soluble derivatives at the same conditions, so they were also selected for microalgae pretreatment.

### 3.5 Application of CLEAs and Combi-CLEAs on Microalgae Pretreatment to Break Cell Wall

As a proof of concept, on the basis of the previous works on US-assisted microalgae pretreatment and extraction (Bautista et al., 2015; Sánchez-Bayo et al., 2019; Sheldon, 2019; Blanco-Llamero et al., 2021), enzymatic reactions were carried out with optimum CLEAs and combi-CLEAs (using ethanol at 90%, acetone at 90%, and PEG at 80%) combined with US and compared to soluble Alcalase®, Viscozyme®, and Celluclast® also combined with US. All preparations could be compared in terms of oil yield because they had the same amount of enzyme. A blank test without biocatalyst was conducted to observe US effects on *N. gaditana* cell wall. Results were also compared with untreated microalgal oil recovery.

**FIGURE 6:**
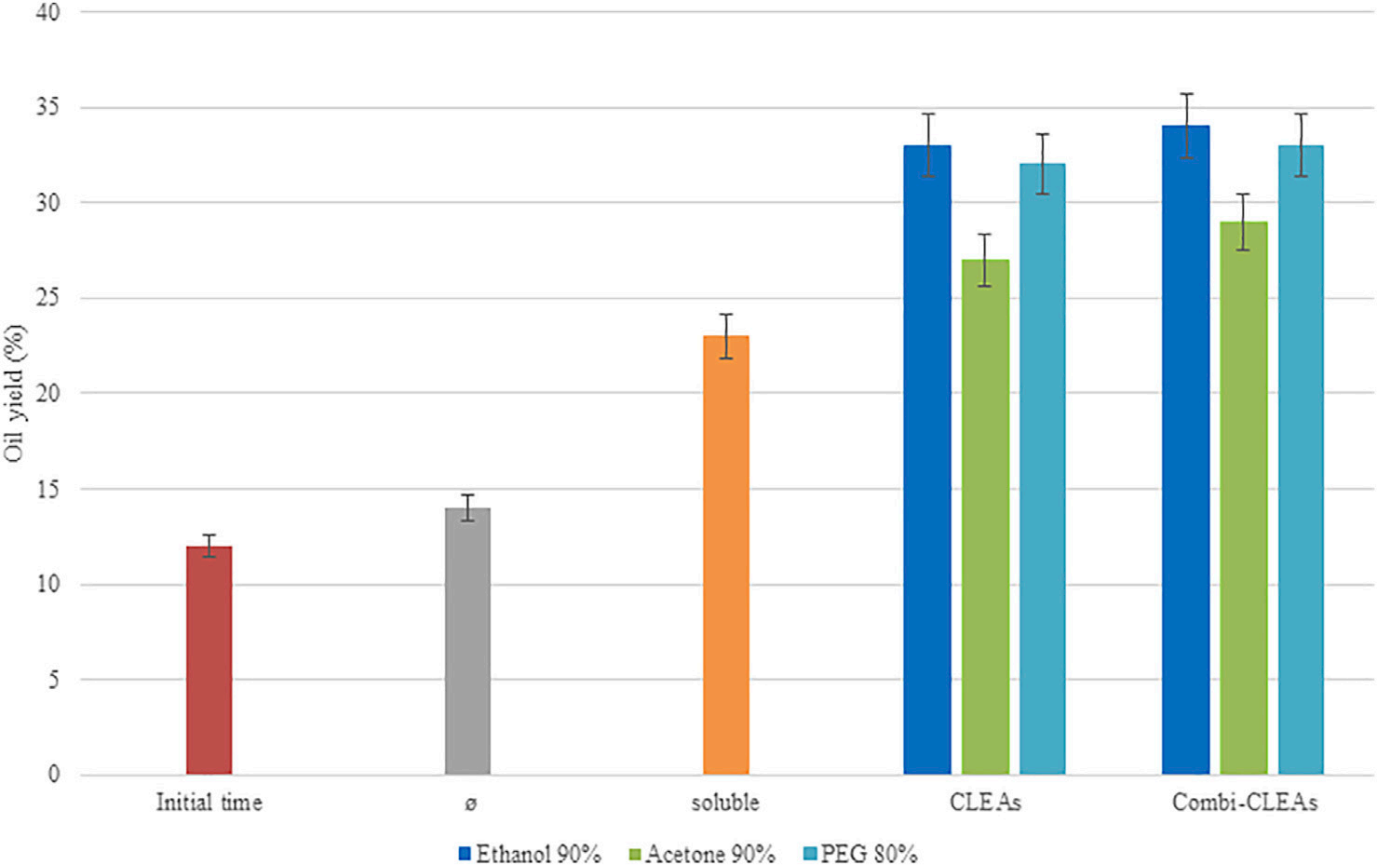
Oil yields (%) obtained after enzymatic pretreatment of *N. gaditana* biomass at 55°C and pH 5.0 during 6 h in an US bath using soluble enzymes, optimal CLEAs, or optimal combi-CLEAs. Results were compared to initial time (unpretreated biomass) and microalgae biomass incubated in the US bath without enzymes (Ø).

In Figure 6, it should be pointed out that the oil yield of the microalgae extraction slightly increased when the US bath was used on its own (14.56%) compared with the initial time (12.02%). However, remarkably, higher results were obtained when enzymes in combination were added to the process (23.65%), doubling the results obtained by initial time (*p* < 0.05), demonstrating the effect of the enzymes in the cell wall breakage and suggesting the potential of this combined pretreatment process.

Oil yields obtained for CLEAs ranged from 27.15% to 33.23%, whereas the results obtained when the combi-CLEAs were employed varied from 29.02% to 34.11%. Interestingly, when the derivatives were applied to the pretreatment process, the immobilized enzymes, either employing CLEAs or combi-CLEAs, maintained the promising results obtained by the soluble enzymes and they even improved, which may be due to the remarkably higher stability of the derivatives described before. Indeed, the interaction between enzymes and substrate related to combi-CLEAs and microalgae was satisfactory as the derivatives were in the form of aggregates and the microalgae solution was able to enter and interact with the enzymes avoiding mass transfer problems. On the other hand, regarding to the combi-CLEAs, even the difficulties of their production due to the different interactions of the structures of the enzyme in the aggregate, they were obtained satisfactory in the present work, and it is important to highlight the effect of the combi-CLEAs on the microalgae pretreatment. The improvement compared to the soluble enzymes and the CLEAs of each enzyme separated is that this process can obtain all the reactions in one pot within the same aggregate. As it can be seen in Figure 6, the best biocatalyst in terms of oil yield obtained was the one produced under the conditions of ethanol at 90%, either combi-CLEA or CLEA. Indeed, the similarity between the results of CLEAs and combi-CLEAs showed the ability of the biocatalytic process, allowing the transference between the microalgae and the aggregate, suggesting that the enzymes are accessible enough when the three enzymes were combined in one combi-CLEA. This fact supports the production of combi-CLEA, because they maintained the same results and reduce the operational steps, cost, and time of production process.

As a perspective of future, once the effectiveness of combi-CLEAs was proved, especially the ones of ethanol at 90%, it will be interesting to produce magnetic combi-CLEAs that make it possible to recover the biocatalyst and recycle it in different cycles of microalgae pretreatment, making the process even more efficient in terms of energy, time, material and economics.

### 3.6 Structural Analysis by Scanning Electron Microscopy

**FIGURE 7:**
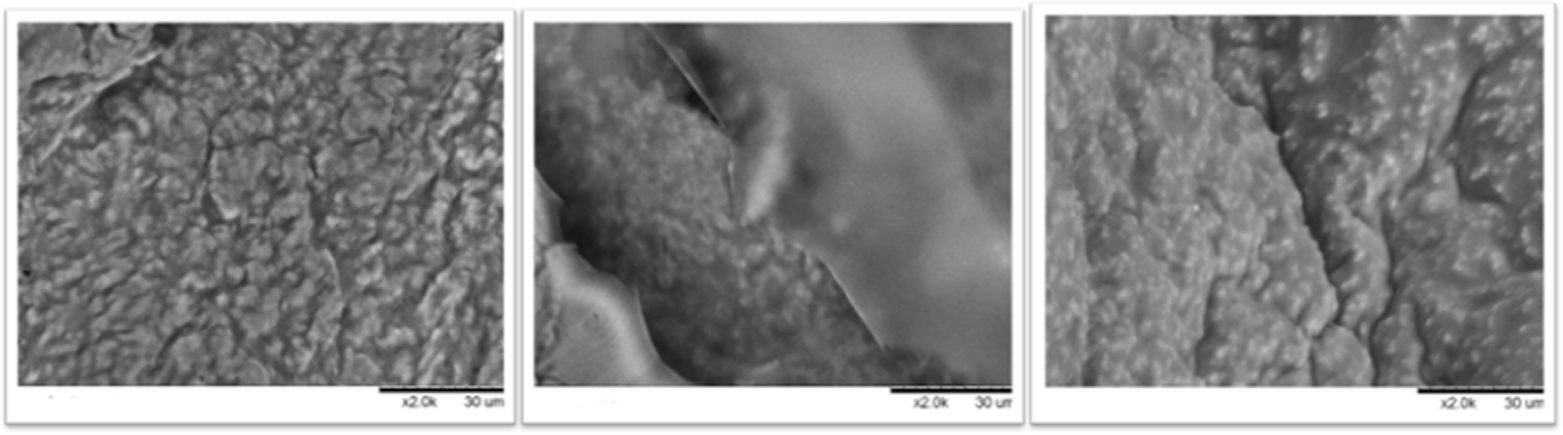
Combi-CLEAs studied by SEM: ethanol at 90% in the left, acetone at 90% in the middle, and PEG at 80% in the right.

**FIGURE 8:**
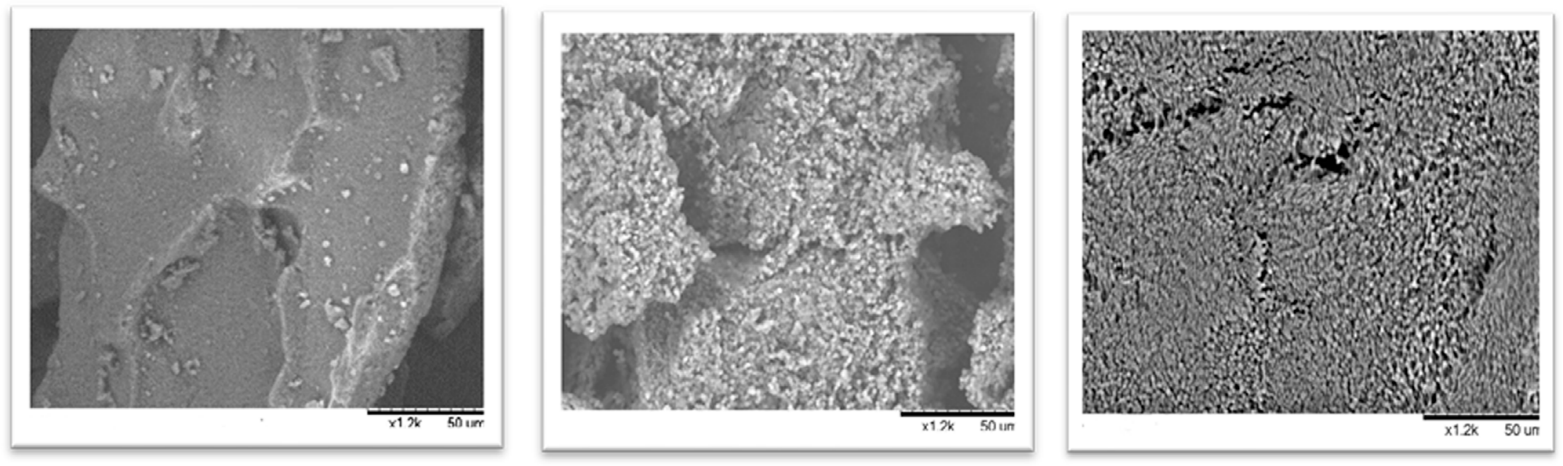
*N. gaditana* biomass studied by SEM: initial time, up and left; pretreated biomass using soluble enzymes, up and right; and pretreated biomass using CLEAs ethanol at 90%, down.

To study morphologic details of the surface of the derivatives, SEM was employed to study the best combi-CLEAs obtained and the microalgae cell wall to study disruption rate and confirm the effectiveness of the method developed. Combi-CLEAs using PEG at 80% and ethanol and acetone at 90% were studied (Figure 7) and compared to the method using soluble enzymes and with the initial time of the intact biomass (Figure 8).

As it can be seen in Figure 7, microalgae cell wall appeared to be broken in great degree using all the combi-CLEAs for the enzymatic pretreatment of *Nannochloropsis gaditana* cell wall. Furthermore, compared with the initial time, the structure of the biomass was clearly damaged. In both cases, employing either soluble enzymes or carrier-free derivatives, the microalgal cell wall was ruined. These results confirmed the effectiveness of the CLEAs, breaking the microalgal cell wall and their action on microalgae, which is in agreement with the oil yield results obtained from the reactions previously described. Remarkably, microalgae could easily penetrate inside the CLEAs because it was prepared in the form of carrier-free derivatives, avoiding mass transfer difficulties that could happen using traditional derivatives immobilized inside the porous structure of traditional activated supports.

## 4 Conclusion

In this work, CLEAs and combi-CLEAs of Alcalase®, Viscozyme®, and Celluclast® from commercial enzymes solutions for biomass pretreatment are produced and characterized. As relevant results, CLEAs of the three enzymes were successfully produced with high immobilization rates (over 80%). Interesting immobilization results were in agreement with the stability test results that were obtained by incubating the derivatives at different conditions and by the results of the proof-of-concept reactions on microalgal biomass. On the other hand, the best-obtained conditions showed its usefulness in the pretreatment of microalgae, facilitating the cell disruption and reducing possible variations due to the instability of soluble enzymes, as it is shown in the increased extraction yield and in the structural morphology of the cell wall by SEM.

As a conclusion, the results obtained show the application of CLEAs as a promising technology for microalgae pretreatment to improve the stability of enzymes commonly used in this biomass and reduce the amount of solvents and energy employed in the subsequent extraction, increasing extraction yield and reducing environmental pollution and global cost of the process (Sheldon, 2019).

## Data Availability

The original contributions presented in the study are included in the article/supplementary material; further inquiries can be directed to the corresponding authors.

## References

[B1] Bautista L. F. Vicente G. Mendoza Á. González S. Morales V. (2015). Enzymatic Production of Biodiesel From Nannochloropsis Gaditana Microalgae Using Immobilized Lipases in Mesoporous Materials. Energy Fuels. 29, 4981–4989. 10.1021/ef502838h

[B2] Bilal M. Noreen S. Asgher M. Parveen S. (2021). Development and Characterization of Cross-Linked Laccase Aggregates (Lac-CLEAs) From *Trametes versicolor* IBL-04 as Ecofriendly Biocatalyst for Degradation of Dye-Based Environmental Pollutants. Environ. Technology Innovation. 21, 101364. 10.1016/j.eti.2021.101364

[B3] Blanco-Llamero C. García-García P. Señoráns F. J. (2021). Combination of Synergic Enzymes and Ultrasounds as an Effective Pretreatment Process to Break Microalgal Cell Wall and Enhance Algal Oil Extraction. Foods. 10, 1928. 10.3390/foods10081928 34441705 PMC8392219

[B4] Blanco-Llamero C. Señoráns F. J. (2021). Biobased Solvents for Pressurized Liquid Extraction of Nannochloropsis Gaditana Omega-3 Lipids. Mar. Drugs. 19, 107. 10.3390/md19020107 33673060 PMC7918423

[B5] Bradford M. M. (1976). A Rapid and Sensitive Method for the Quantitation of Microgram Quantities of Protein Utilizing the Principle of Protein-Dye Binding. Anal. Biochem. 72, 248–254. 10.1006/abio.1976.9999 942051

[B6] Castejón N. Luna P. Señoráns F. J. (2018). Alternative Oil Extraction Methods from *Echium plantagineum* L. Seeds Using Advanced Techniques and Green Solvents. Food Chem. 244, 75–82. 10.1016/j.foodchem.2017.10.014 29120808

[B7] Castejón N. Señoráns F. J. (2019). Simultaneous Extraction and Fractionation of Omega-3 Acylglycerols and Glycolipids From Wet Microalgal Biomass of Nannochloropsis Gaditana Using Pressurized Liquids. Algal Res. 37, 74–82. 10.1016/j.algal.2018.11.003

[B8] Castejón N. Señoráns F. J. (2020). Enzymatic Modification to Produce Health-Promoting Lipids From Fish Oil, Algae and Other New Omega-3 Sources: A Review. New Biotechnol. 57, 45–54. 10.1016/j.nbt.2020.02.006 32224214

[B9] Chen Q. Liu D. Wu C. Xu A. Xia W. Wang Z. (2017). Influence of a Facile Pretreatment Process on Lipid Extraction from Nannochloropsis Sp. Through an Enzymatic Hydrolysis Reaction. RSC Adv. 7 (84), 53270–53277. 10.1039/c7ra11483d

[B10] Chen Q. Liu D. Wu C. Yao K. Li Z. Shi N. (2018). Co-Immobilization of Cellulase and Lysozyme on Amino-Functionalized Magnetic Nanoparticles: An Activity-Tunable Biocatalyst for Extraction of Lipids From Microalgae. Bioresour. Technology. 263 (May), 317–324. 10.1016/j.biortech.2018.04.071 29753933

[B11] Dixon C. Wilken L. R. (2018). Green Microalgae Biomolecule Separations and Recovery. Bioresour. Bioproc. 5, 1. 10.1186/s40643-018-0199-3

[B12] Folch J. Lees M. Stanley G. H. S. (1957). A Simple Method for the Isolation and Purification of Total Lipides From Animal Tissues. J. Biol. Chem. 226 (1), 497–509. 10.1016/s0021-9258(18)64849-5 13428781

[B13] Gao S. Rojas-Vega F. Rocha-Martin J. Guisán J. M. (2021). Oriented Immobilization of Antibodies through Different Surface Regions Containing Amino Groups: Selective Immobilization Through the Bottom of the Fc Region. Int. J. Biol. Macromolecules. 177, 19–28. 10.1016/j.ijbiomac.2021.02.103 33607135

[B14] García-García P. Fernandez-Lorente G. Guisan J. M. (2021). Capture of Enzyme Aggregates by Covalent Immobilization on Solid Supports. Relevant Stabilization of Enzymes by Aggregation. J. Biotechnol. 325, 138–144. 10.1016/j.jbiotec.2020.11.006 33249106

[B15] Gong M. Bassi A. (2016). Carotenoids From Microalgae: A Review of Recent Developments. Biotechnol. Adv. 34 (8), 1396–1412. 10.1016/j.biotechadv.2016.10.005 27816618

[B16] Guajardo N. Ahumada K. Domínguez de María P. (2021). Immobilization of Pseudomonas Stutzeri Lipase Through Cross-Linking Aggregates (CLEA) for Reactions in Deep Eutectic Solvents. J. Biotechnol. 337, 18–23. 10.1016/j.jbiotec.2021.06.021 34171440

[B17] Hero J. S. Morales A. H. Perotti N. I. Romero C. M. Martinez M. A. (2020). Improved Development in Magnetic Xyl-CLEAs Technology for Biotransformation of Agro-Industrial By-Products Through the Use of a Novel Macromolecular Cross-Linker. Reactive Funct. Polym. 154, 104676. 10.1016/j.reactfunctpolym.2020.104676

[B18] Katiyar R. Arora A. (2020). Health Promoting Functional Lipids From Microalgae Pool: A Review. Algal Res. 46, 101800. 10.1016/j.algal.2020.101800

[B19] Kimberle P. d. S. Carolina M.-S. Ana I. S. B. Luciana R. B. G. (2020). Modifying Alcalase Activity and Stability by Immobilization onto Chitosan Aiming at the Production of Bioactive Peptides by Hydrolysis of tilapia Skin Gelatin. Process Biochem. 97, 27–36. 10.1016/j.procbio.2020.06.019

[B21] Mahdy A. Mendez L. Ballesteros M. González-Fernández C. (2014). Enhanced Methane Production of Chlorella Vulgaris and Chlamydomonas Reinhardtii by Hydrolytic Enzymes Addition. Energ. Convers. Management. 85, 551–557. 10.1016/j.enconman.2014.04.097

[B22] Perwez M. Mazumder J. A. Noori R. Sardar M. (2021). Magnetic Combi CLEA for Inhibition of Bacterial Biofilm: A Green Approach. Int. J. Biol. Macromolecules. 186, 780–787. 10.1016/j.ijbiomac.2021.07.091 34280443

[B23] Poorakbar E. Shafiee A. Saboury A. A. Rad B. L. Khoshnevisan K. Ma'mani L. (2018). Synthesis of Magnetic Gold Mesoporous Silica Nanoparticles Core Shell for Cellulase Enzyme Immobilization: Improvement of Enzymatic Activity and thermal Stability. Process Biochem. 71, 92–100. 10.1016/j.procbio.2018.05.012

[B24] Punia S. Sandhu K. S. Siroha A. K. Dhull S. B. (2019). Omega 3-Metabolism, Absorption, Bioavailability and Health Benefits-A Review. PharmaNutrition. 10, 100162. 10.1016/j.phanu.2019.100162

[B25] Romero O. Guisán J. M. Illanes A. Wilson L. (2012). Reactivation of Penicillin Acylase Biocatalysts: Effect of the Intensity of Enzyme-Support Attachment and Enzyme Load. J. Mol. Catal. B: Enzymatic. 74, 224–229. 10.1016/j.molcatb.2011.10.009

[B26] Romero-Fernández M. Moreno-Perez S. H. Orrego A. Martins de Oliveira S. I. Santamaría R. Díaz M. (2018). Designing Continuous Flow Reaction of Xylan Hydrolysis for Xylooligosaccharides Production in Packed-Bed Reactors Using Xylanase Immobilized on Methacrylic Polymer-Based Supports. Bioresour. Technology. 266, 249–258. 10.1016/j.biortech.2018.06.070 29982045

[B27] Safi C. Olivieri G. Campos R. P. Engelen-Smit N. Mulder W. J. van den Broek L. A. M. (2017). Biorefinery of Microalgal Soluble Proteins by Sequential Processing and Membrane Filtration. Bioresour. Technology. 225, 151–158. 10.1016/j.biortech.2016.11.068 27888732

[B28] Sánchez-Bayo A. Morales V. Rodríguez R. Vicente G. Bautista L. F. (2019). "Biodiesel Production (FAEEs) by Heterogeneous Combi-Lipase Biocatalysts Using Wet Extracted Lipids From Microalgae. Catalysts. 9, 296. 10.3390/catal9030296

[B29] Señoráns M. Castejón N. Señoráns F. J. (2020). Advanced Extraction of Lipids with DHA From *Isochrysis galbana* with Enzymatic Pre-Treatment Combined With Pressurized Liquids and Ultrasound Assisted Extractions. Molecules. 25 (14), 3310. 10.3390/molecules25143310 PMC7397065 32708275

[B31] Sheldon R. (2019). CLEAs, Combi-CLEAs and 'Smart' Magnetic CLEAs: Biocatalysis in a Bio-Based Economy. Catalysts. 9, 26. 10.3390/catal9030261

[B30] Velasco-Lozano S. (2020). Immobilization of Enzymes as Cross-Linked Enzyme Aggregates: General Strategy to Obtain Robust Biocatalysts. Methods Mol. Biol. 2100, 345–361. 10.1007/978-1-0716-0215-7_23 31939135

[B32] Wang Q. Xie Y. Johnson D. R. Li Y. He Z. Li H. (2019). Ultrasonic‐Pretreated Lipase‐catalyzed Synthesis of Medium-Long-Medium Lipids Using Different Fatty Acids as Sn ‐2 Acyl‐Site Donors. Food Sci. Nutr. 7 (7), 2361–2373. 10.1002/fsn3.1083 31367365 PMC6657711

[B33] Xu M. Ji D. Deng Y. Agyei D. (2020). Preparation and Assessment of Cross-Linked Enzyme Aggregates (CLEAs) of β-Galactosidase From Lactobacillus Leichmannii 313. Food Bioproducts Process. 124, 82–96. 10.1016/j.fbp.2020.08.004

[B34] Yen H.-W. Hu I.-C. Chen C.-Y. Ho S.-H. Lee D.-J. Chang J.-S. (2013). Microalgae-Based Biorefinery - From Biofuels to Natural Products. Bioresour. Technology. 135, 166–174. 10.1016/j.biortech.2012.10.099 23206809

[B35] Zhang R. Chen J. Zhang X. (2018a). Extraction of Intracellular Protein From Chlorella Pyrenoidosa Using a Combination of Ethanol Soaking, Enzyme Digest, Ultrasonication and Homogenization Techniques. Bioresour. Technology. 247, 267–272. 10.1016/j.biortech.2017.09.087 28950135

[B36] Zhang W. Leong S. M. Zhao F. Zhao F. Yang T. Liu S. (2018b). Viscozyme L Pretreatment on Palm Kernels Improved the Aroma of palm Kernel Oil After Kernel Roasting. Food Res. Int. 107, 172–181. 10.1016/j.foodres.2018.02.023 29580475

[B37] Zhang T.-T. Xu J. Wang Y.-M. Xue C.-H. (2019). Health Benefits of Dietary Marine DHA/EPA-Enriched Glycerophospholipids. Prog. Lipid Res. 75, 100997. 10.1016/j.plipres.2019.100997 31442526

[B38] Zhang W. Zhao F. Yang T. Zhao F. Liu S. (2017). Celluclast 1.5L Pretreatment Enhanced Aroma of Palm Kernels and Oil After Kernel Roasting. J. Sci. Food Agric. 97 (15), 5146–5157. 10.1002/jsfa.8394 28436034

[B39] Zuorro A. Maffei G. Lavecchia R. (2016). Optimization of Enzyme-Assisted Lipid Extraction From Nannochloropsis Microalgae. J. Taiwan Inst. Chem. Eng. 67, 106–114. 10.1016/j.jtice.2016.08.016

